# Digital Divide or Compensatory Dividend? The Longitudinal Impact of Smart Health Monitoring on Intrinsic Capacity Trajectories

**DOI:** 10.3390/healthcare14152254

**Published:** 2026-07-23

**Authors:** Bin Zhang, Ying Liu, Shanna Li, Linlin Zhang, Lin Guo

**Affiliations:** 1 School of Economics and Management, Weinan Normal University, Chaoyang Middle Road, Linwei District, Weinan 714099, China; popeye0416@wnu.edu.cn; 2 School of Humanities and Management, Zhejiang Chinese Medical University, No. 260 Baichuan Street, Fuyang District, Hangzhou 311402, China; 20241162@zcmu.edu.cn; 3 School of Management, Shandong Second Medical University, No. 7166, Baotong West Street, Weifang 261053, China; lishanna@sdsmu.edu.cn

**Keywords:** intrinsic capacity, digital health, latent profile analysis, profile transitions, aging trajectories

## Abstract

**Background:** The preservation of intrinsic capacity (IC) is a global public health priority, and digital health technologies offer promising avenues for managing functional decline. However, the longitudinal impact of specific smart monitoring devices on IC trajectories remains underexplored, and theoretical debates persist regarding whether digital interventions exacerbate health inequalities (the “digital divide”) or mitigate them (the “compensatory effect”). **Methods:** Using a balanced panel of 2432 older adults from the China Longitudinal Aging Social Survey (CLASS 2020–2023), we conducted wave-specific latent profile analyses based on five standardized WHO intrinsic-capacity domains. Modal class assignment and cross-wave profile matching were subsequently used to describe profile stability and transitions. The longitudinal protective association of baseline smart blood pressure (BP) monitor use with IC trajectories was evaluated using multinomial logistic regression with Inverse Probability Weighting (IPW). Exploratory structural path analysis was conducted to examine a potential mediating pathway. **Results:** The baseline latent profile analysis identified three profiles: Robust Capacity (80.2%), Cognitively Impaired (10.9%), and Sensory Impaired (8.8%). The Sensory Impaired class exhibited strong path dependence, with 62.79% of participants remaining in the same state over three years. Baseline estimates revealed that smart BP monitor usage was significantly associated with a reduced relative risk of transitioning into or remaining in the Sensory Impaired profile (RRR = 0.557, 95% CI: 0.325–0.955). The IPW analysis yielded a directionally consistent estimate (RRR = 0.696), although the association was attenuated and was not statistically significant (*p* = 0.162). Subgroup and pathway analyses yielded non-significant but suggestive findings, indicating that protective associations might be concentrated among vulnerable groups, particularly women and individuals with lower educational attainment. **Conclusions:** Smart physiological monitoring interventions demonstrate a longitudinal protective association against the persistence of physical frailty. Rather than definitively bridging the digital divide, targeted medical-grade digital tools may serve as compensatory resources for specific vulnerable subpopulations. These observational associations highlight the need for further quasi-experimental testing before integrating specific digital interventions into community-based chronic care ecosystems.

## 1. Introduction

The preservation of intrinsic capacity (IC)—defined as the composite of physical and mental capacities—is central to mitigating the health and economic burdens associated with global population aging [1,2]. Concurrently, digital health technologies, particularly smart monitoring devices and Internet of Things (IoT) applications, have rapidly emerged as prominent tools for managing chronic conditions and facilitating independent living among older adults [3,4]. The prevailing narrative within the gerontological and public health literature suggests that digital interventions, such as electronic blood pressure (BP) monitoring, yield substantial clinical benefits by enabling timely medical responses and enhancing long-term medication adherence [5]. Consequently, the integration of smart healthcare into community settings is increasingly viewed as an essential public health imperative to optimize functional longevity [6,7].

Despite these technological advancements, a significant theoretical conflict persists regarding the distribution and efficacy of the “digital health dividend.” A substantial body of literature warns of a broader “digital divide” capable of triggering a Matthew effect, wherein digital technologies may disproportionately benefit individuals with higher socioeconomic status and baseline digital literacy, thereby exacerbating existing social and health disparities [8,9]. Conversely, an emerging perspective proposes a “compensatory effect.” Recent empirical studies indicate that certain digital infrastructures and targeted eHealth interventions might disproportionately benefit highly vulnerable subgroups—such as those with lower educational attainment or compromised baseline health—by compensating for limitations in traditional healthcare resources [10,11,12]. The structural conditions and specific technological modalities under which digital tools operate as equalizers rather than stratifiers remain insufficiently conceptualized within the current literature.

Methodologically, evaluating the longitudinal impact of digital health interventions on IC is constrained by two primary limitations. First, many existing studies still operationalize functional decline using continuous or trajectory-based measures, which may not fully capture the complex, co-occurring nature of multidimensional impairments [13,14]. The path dependence associated with specific combinations of impairments—such as transitions from robust health to concurrent sensory and locomotor deficits—requires advanced modeling to quantify state stability and individual-level vulnerability [15,16,17]. Second, the clinical efficacy of digital devices is frequently evaluated without a rigorous examination of the underlying biological pathways. The mechanism through which technological adoption translates into preserved IC is often treated as a “black box,” with limited empirical validation of intermediate stabilizing factors, such as the stabilization of multimorbidity burden [18,19,20].

To address these empirical and methodological gaps, this study uses a latent profile transition framework, followed by multinomial logistic regression with Inverse Probability Weighting (IPW) and structural path analysis, drawing on longitudinal data from the China Longitudinal Aging Social Survey (CLASS 2020–2023). Moving beyond descriptive analysis, this study tests the following five theory-informed hypotheses derived from prevailing gerontological and sociological frameworks:

**Hypothesis** **1.** *(Latent Profiles): Older adults will exhibit distinct, multidimensional latent profiles of intrinsic capacity rather than a uniform continuum of decline, specifically differentiating cognitive and sensory/physical vulnerabilities.*

**Hypothesis** **2.** *(Transition Patterns): Intrinsic capacity trajectories will demonstrate strong path dependence. Specifically, impaired latent profiles will exhibit high longitudinal stability and limited natural reversibility over the three-year tracking period compared to the robust state.*

**Hypothesis** **3.** *(Protective Association): The baseline use of targeted smart physiological monitoring devices (e.g., smart BP monitors) will be prospectively associated with a reduced relative risk of transitioning into or remaining in impaired capacity profiles.*

**Hypothesis** **4.** *(Mediating Mechanism): The protective longitudinal association between smart BP monitor use and capacity transitions will be mediated by a “biological buffer”—specifically, the stabilization of the multimorbidity (chronic disease) burden over time.*

**Hypothesis** **5.** *(Heterogeneity and Compensatory Dividend): In alignment with the compensatory effect theory, the protective association of digital monitoring will exhibit structural heterogeneity. We hypothesize that the digital health dividend will be more pronounced among sociodemographically disadvantaged subpopulations (e.g., those with lower educational attainment, rural residents, or specific gender or age groups) who traditionally face barriers to conventional healthcare resources.*

To directly operationalize the theoretical tension embedded within Hypothesis 5, we further specify two competing distributional expectations.

**Hypothesis** **5a.** *(Matthew-Effect Expectation): If the digital divide predominates, the protective association of smart physiological monitoring will be more pronounced among socioeconomically advantaged and digitally connected older adults, such as those with higher educational attainment, urban residence, or greater digital access, thereby potentially widening existing health disparities.*

**Hypothesis** **5b.** *(Compensatory-Effect Expectation): If the compensatory effect predominates, the protective association will be more pronounced among socioeconomically or clinically vulnerable older adults, such as those with lower educational attainment, limited conventional healthcare resources, or greater baseline vulnerability, thereby potentially narrowing existing health disparities.*

These competing expectations concern the distribution of the association across social groups and are analytically distinct from Hypothesis 4. Hypothesis 4 evaluates a potential clinical pathway through the stabilization of chronic disease burden, whereas Hypothesis 5 evaluates whether the magnitude of the association varies according to socio-demographic position. The compensatory effect is therefore not assumed to operate exclusively through the proposed biological buffer. It may also arise through separate or complementary pathways, including substitution for limited healthcare resources, more timely health feedback, enhanced healthcare engagement, or family and community support.

By testing these explicit hypotheses, this study aims to systematically quantify the longitudinal stability of capacity loss and evaluate the precise structural conditions under which digital tools operate as equalizers rather than stratifiers, offering critical implications for the design of equitable digital health policies [21].

## 2. Materials and Methods

### 2.1. Data Sources and Sample Selection

This study utilizes longitudinal data from the 2020 and 2023 waves of the China Longitudinal Aging Social Survey (CLASS), a nationally representative, biennial survey that tracks the socioeconomic and health status of the aging population in China. The CLASS database provides a robust framework for assessing intrinsic capacity (IC) and health behaviors, employing standardized interview protocols administered by trained enumerators across diverse provinces.

To construct a consistent longitudinal analytical cohort for the cross-wave profile-transition analysis, a rigorous case-wise sample-selection procedure was implemented. We sequentially excluded non-longitudinal respondents (i.e., those lost to follow-up, deceased, or newly recruited in the 2023 wave) and respondents with item non-response on any focal variable or baseline covariate. Following this strict person-level listwise deletion protocol, the final analytical sample comprised a balanced panel of 2432 unique older adults (4864 person-wave observations). A comprehensive sample attrition flowchart is provided in Table S1, documenting the alignment between baseline tracking rows and the final balanced analytical cohort. Given the substantial attrition during construction of the final analytical cohort, baseline characteristics were compared between the 2432 respondents included in the final analysis and the 8963 baseline respondents who were not retained because of loss to follow-up, death before follow-up, or incomplete information on the study variables (Supplementary Table S6). Significant differences were observed in age, rural residence, educational attainment, internet use, chronic disease burden, and baseline cognition, whereas no statistically significant differences were observed in gender, personal income, or social-network activity. These findings indicate that selection into the final analytical cohort was socially and clinically patterned. For item-level missingness, the complete-case analysis was conducted under a missing-at-random assumption conditional on the observed covariates. However, this assumption cannot be empirically verified, and adjustment for observed characteristics cannot fully eliminate selection bias associated with loss to follow-up, mortality, or incomplete data.

### 2.2. Variable Definitions

Intrinsic capacity (IC) was operationalized according to the WHO framework, encompassing five interconnected domains: cognition, psychological capacity, sensory capacity, locomotor capacity, and vitality. In this study, cognition was assessed using cognitive performance indicators; psychological capacity was evaluated using psychological functioning indicators; sensory capacity was measured based on vision- and hearing-related functions; locomotor capacity was assessed through mobility-related functional measures; and vitality was represented by body mass index (BMI). Detailed item content, scoring procedures, coding directions, and score construction for each intrinsic-capacity domain and smart-device variable are provided in Supplementary Table S2. All five IC domain indicators were standardized before latent profile analysis to ensure comparability across different measurement scales. Briefly, the primary dependent variable is the latent health profile membership derived from the 2023 follow-up wave, constructed using the same intrinsic capacity (IC) indicators as those used at baseline. The core independent variable is the binary indicator of smart health device use at baseline (Wave 2020), specifically capturing the use of electronic blood pressure monitors, blood lipid testers, smart wristbands, and other assistive technologies. Control variables encompass a robust set of socio-demographic factors—including age, gender, urban residency, marital status, education level, household size, and personal income—alongside health-related covariates such as multimorbidity burden (chronic conditions count), internet usage habits, and social network scores. All continuous financial and physical metrics were standardized or log-transformed as appropriate to ensure model stability. For the intrinsic capacity modeling, the raw scores of the five domains were standardized into Z-scores. All indicators were strictly coded in a positive direction, meaning that higher Z-scores consistently reflect better functional capacity across all dimensions.

### 2.3. Analytic Strategy

**Figure 1:**
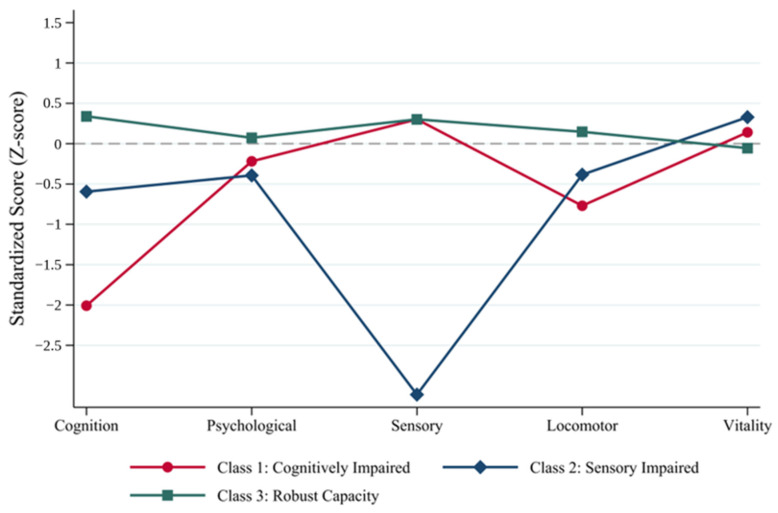
Latent Profiles of Baseline Intrinsic Capacity Based on Standardized Z-scores. Notes: The y-axis represents the standardized Z-scores for each intrinsic capacity domain, which account for differences in measurement scales across indicators. The dashed horizontal gray line at zero indicates the overall sample mean. Class 1 (Cognitively Impaired, red line) exhibits a pronounced deficit specifically in cognitive function. Class 2 (Sensory-Impaired, blue line) is characterized by severe sensory limitations and concurrent moderate locomotor decline. Class 3 (Robust Capacity, green line) represents the majority of the cohort, with scores consistently near or slightly above the sample average across all physiological and psychological domains.

Our analysis follows a five-stage sequential framework to establish robust longitudinal evidence:

(1) Latent Profile Analysis (LPA): We utilized the five standardized IC indicators at baseline (Wave 2020) to identify unobserved health states. Models were estimated with robust standard errors. To reduce the risk of local maxima and improve convergence stability, we specified stringent expectation-maximization (EM) iteration settings (emopts (iterate(100)) iterate(200)). Although information criteria (AIC/BIC) slightly favored the 4-class model, its classification quality deteriorated substantially (Entropy dropped to 0.750), and it produced a fragmented latent class comprising only 1.81% of the sample, failing to meet minimum class-size criteria indicating model over-extraction. Therefore, the 3-class model was selected as the optimal taxonomy, balancing empirical fit, high classification quality (Entropy = 0.941), and theoretical interpretability (see Figure 1 for the visualized class characteristics and Table S3 for the full enumeration process). Following class identification, individuals were assigned to their most likely latent profile using modal assignment based on maximum posterior probabilities.
(2) Cross-Wave Profile-Transition Analysis: To describe changes in intrinsic-capacity profiles from 2020 to 2023, we used a classify-then-analyze profile-transition framework. A latent profile model was fitted separately at each wave using the same five standardized intrinsic-capacity domains. Participants were assigned to their most likely profile at each wave using modal assignment based on maximum posterior probabilities. The wave-specific profiles were then matched across waves according to their substantive indicator patterns, and cross-wave transition proportions were calculated.

This approach differs from a jointly estimated full-information latent transition model; longitudinal measurement invariance was neither formally tested nor imposed. Accordingly, the resulting matrix should be interpreted as describing observed cross-wave transition proportions between modally assigned wave-specific profiles, rather than model-estimated latent transition probabilities.

(3) Confounding Adjustment via IPW-Logit: To evaluate the “digital health dividend,” we employed multinomial logistic regression. To mitigate endogeneity arising from self-selection bias—whereby baseline health-conscious individuals are more likely to adopt digital tools—we utilized Inverse Probability Weighting (IPW) to balance observed covariates between users and non-users, thereby reducing observable selection bias.
(4) IPW Implementation: To mitigate observable selection bias, we implemented Inverse Probability Weighting (IPW). A multivariable logistic regression was used to calculate the propensity scores for smart BP monitor use, conditioning on sociodemographic variables (age, gender, urban residency, marital status, education, log-income), health needs (chronic disease count), and digital literacy (internet use). Unstabilized Average Treatment Effect (ATE) weights were then generated. The 99th percentile of the unstabilized weights was 5.21, indicating no pronounced weight instability; therefore, no trimming was applied. Covariate balance was assessed using Standardized Mean Differences (SMDs), with all covariates achieving SMDs below 0.10 post-weighting (detailed in Supplementary Table S4). Robust sandwich standard errors were employed in the final weighted regression.
(5) Robustness and Mechanism Validation: We verified findings through alternative model specifications and performed structural path analysis to examine whether the longitudinal stabilization of multimorbidity burden mediated the association between digital monitoring and health trajectories.

To explicitly link the theoretical framework to the empirical design, Hypothesis 4 was examined through the exploratory KHB pathway analysis assessing whether changes in chronic disease burden accounted for part of the association between baseline smart BP monitor use and subsequent intrinsic-capacity profiles. The competing expectations under Hypothesis 5 were evaluated through subgroup-specific models and formal interaction terms between smart BP monitor use and gender, education, residence, and age group. A stronger association among relatively advantaged groups would be more consistent with the Matthew-effect expectation, whereas a stronger association among disadvantaged or vulnerable groups would be more consistent with the compensatory-effect expectation. Because statistical significance within one subgroup does not establish between-group heterogeneity, support for either perspective was evaluated primarily through the direction and statistical evidence of the formal interaction terms.

## 3. Results

### 3.1. Baseline Characteristics and Latent Profiles

Based on the Latent Profile Analysis (LPA) of the baseline standardized intrinsic capacity Z-scores, the analytical sample was classified into three distinct health states: Robust Capacity (80.2%), Cognitively Impaired (10.9%), and Sensory Impaired (8.8%) (Table 1). It is crucial to note that the emergence of these specific impaired profiles was entirely data-driven rather than theoretically pre-imposed. The LPA results demonstrate that early functional decline in this cohort is not a uniform, linear deterioration across all domains. Instead, the model identified two distinct vulnerability pathways. The Robust Capacity group exhibited optimal functional scores across all domains. In contrast, the Cognitively Impaired class presented with severe cognitive deficits (mean = 7.11) and pronounced socioeconomic disadvantages, including the lowest educational attainment (23.31% primary school or below) and marital rates (74.81%). The Sensory Impaired class captured an older, highly vulnerable subpopulation characterized by profound sensory deficits (mean = 0.93) and the heaviest multimorbidity burden (mean chronic conditions = 2.30). This empirical taxonomy strongly aligns with contemporary gerontological evidence indicating that cognitive and sensory declines often act as primary, divergent catalysts for subsequent frailty cascades. Notably, a complex pattern of digital device adoption emerged across the subgroups (see Supplementary Table S5 for the complete baseline distribution of all eight surveyed smart health devices). While the Cognitively Impaired group faced pervasive digital exclusion—evidenced by minimal internet usage (25.19%) and low smart terminal utilization (5.26%)—the Sensory Impaired cohort recorded the highest penetration rates for electronic blood pressure monitors (72.56%) and smart wristbands (22.79%), significantly exceeding the adoption levels observed in the robust majority (*p* < 0.001).

Crucially, while this pattern could partially reflect a compensatory adoption strategy, it must be cautiously interpreted through the lens of confounding by indication (i.e., disease-driven adoption). Given that the Sensory Impaired class concurrently carries the heaviest multimorbidity burden (mean baseline chronic conditions = 2.30), their high utilization of medical-grade monitors is highly likely to be a direct response to pre-existing clinical needs rather than proactive tech-savviness. This alternative explanation underscores the necessity of rigorously controlling for baseline health and chronic disease variables in our subsequent predictive modeling to appropriately isolate the longitudinal associations of the devices.

### 3.2. Cross-Wave Profile Transitions over Three Years

Based on modal profile assignments at the 2020 and 2023 waves, the cross-wave profile-transition matrix is presented in Table 2. The latent profiles demonstrated substantial longitudinal stability, particularly among the healthy majority. Specifically, 97.33% of individuals classified in the Robust Capacity profile in 2020 maintained their robust status in 2023. Conversely, the impaired profiles exhibited substantial persistence and limited reversibility. Among participants assigned to the Sensory Impaired profile at baseline, 62.79% were assigned to the corresponding profile again at follow-up, whereas 30.70% were assigned to the Robust Capacity profile. In contrast, the Cognitively Impaired group demonstrated high state instability, with 87.22% of baseline members transitioning to the Robust Capacity class by 2023. From a clinical perspective, this high rate of apparent ‘recovery’ is counterintuitive for neurodegenerative trajectories. Rather than reflecting true clinical reversal, this transition matrix likely reflects methodological artifacts. First, the strict longitudinal case-wise deletion (Table S1) may have introduced survivor bias, meaning that the most severely impaired individuals likely dropped out, leaving only borderline or mild cases in the analytical cohort. Second, this instability is likely amplified by regression to the mean and classification uncertainty inherent in modal assignment, such that borderline individuals fluctuate across diagnostic thresholds over time. Finally, the cognitive assessment relies on orientation and recall tasks, making follow-up scores highly susceptible to practice effects. Consequently, the trajectory of the Cognitively Impaired class should be interpreted as reflecting measurement instability and survivor bias rather than a genuine 87% clinical recovery rate.

### 3.3. Protective Effects of Smart Health Devices on Capacity Transitions

To investigate whether early adoption of smart health devices was associated with a reduced risk of transitioning into impaired capacity states, a multinomial logistic regression was conducted, with adjustment for baseline latent class membership and sociodemographic covariates (Table 3). With the Robust Capacity class set as the reference outcome, baseline latent profile membership emerged as the strongest predictor of 2023 status, providing evidence of path dependence in physical and cognitive vulnerability (e.g., baseline Sensory Impaired individuals exhibited an RRR of 96.40 for remaining in that class; *p* < 0.001).

Crucially, the analysis revealed specific protective associations consistent with a potential digital health dividend. The baseline use of an electronic blood pressure monitor was significantly associated with a reduced risk of transitioning into or remaining in the Sensory Impaired class by 2023 (RRR = 0.557, 95% CI: 0.325–0.955, *p* = 0.033). This corresponds to a 44.3% lower relative risk of membership in the Sensory Impaired class relative to the Robust Capacity class. This association may reflect more timely medical intervention and improved management of multimorbidity and sensory decline. Other smart devices (e.g., smart wristbands and infrared monitors) did not show statistically significant associations with transitions into the impaired classes (all *p* > 0.05), suggesting that the potential clinical utility of digital interventions for older adults may be device-specific. Among the covariates, advanced age was significantly associated with an increased transition risk across the impaired profiles, while urban residency and higher baseline social network activity were associated with a lower risk of cognitive impairment.

To assess the potential influence of quasi-complete separation, we conducted a supplementary Firth penalized logistic regression for the binary contrast between the Sensory Impaired and Robust Capacity profiles. The estimate for smart BP monitor use remained directionally consistent with the primary model but did not reach conventional statistical significance (OR = 0.605, 95% CI: 0.359–1.020, *p* = 0.059). This sensitivity analysis suggests that the direction of the focal association was not reversed after penalization, although the uncertainty around the estimate remained substantial.

### 3.4. Heterogeneity in the Digital Health Dividend

**Figure 2:**
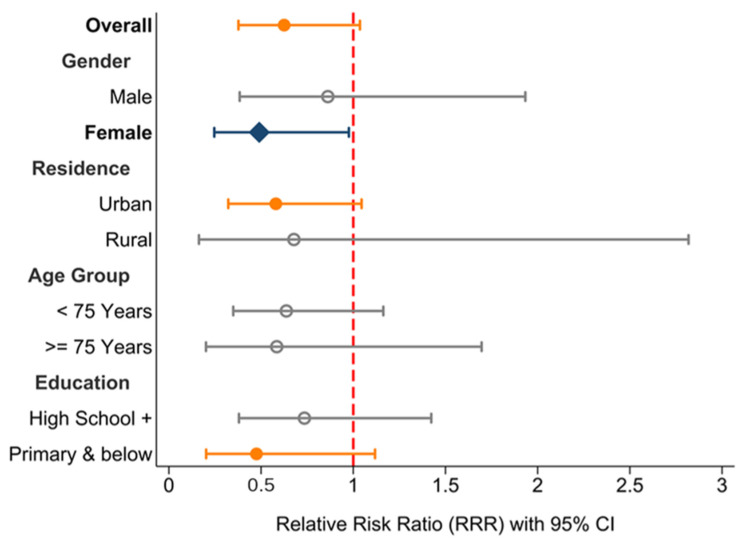
Forest plot of subgroup analyses for the association between smart BP monitor use and transition to the Sensory Impaired profile. Notes: Point estimates represent the Relative Risk Ratios (RRR) derived from multinomial logistic regression models within each respective sub-sample. Horizontal lines indicate 95% Confidence Intervals (CI). The vertical red dashed line denotes the null effect (RRR = 1.0). Estimates situated to the left of the dashed line indicate a lower estimated relative risk of membership in the Sensory Impaired profile relative to the Robust Capacity profile. Color coding and marker shapes reflect statistical significance to ensure accessibility: the dark-blue filled diamond indicates an estimate with a 95% CI excluding 1.0, orange filled circles indicate directionally suggestive estimates, and grey open circles indicate other statistically non-significant estimates. These subgroup analyses should be interpreted as exploratory.

To further unpack the boundary conditions of the digital health dividend, subgroup analyses were conducted. Given that only the electronic BP monitor demonstrated a significant protective association in the baseline exploratory multivariable model testing all eight devices (Table 3), all subsequent heterogeneity, robustness, and mechanism analyses explicitly focused on this targeted device to maintain analytical power and narrative focus. We examined heterogeneity in the association between smart BP monitor use and transition to the Sensory Impaired profile (Figure 2).

The forest plot presents subgroup-specific estimates that suggest possible sociodemographic variation in the association between digital monitoring and subsequent intrinsic-capacity profiles. Descriptive differences were observed between the gender-specific estimates: the protective association of smart BP monitor use was significant among older women (*n* = 1231, RRR = 0.490, 95% CI: 0.246–0.976), whereas the corresponding association was not statistically significant among men (*n* = 1201, RRR = 0.862, 95% CI: 0.384–1.933). Descriptive differences were also observed across residence and educational strata. The protective trend was primarily concentrated among urban residents (*n* = 2130, RRR = 0.580) and those with primary education or below (*n* = 810, RRR = 0.475). The corresponding estimates among rural residents (*n* = 302) and adults aged 75 years or older (*n* = 253) were not statistically significant. Given the small subgroup sizes and limited precision, these findings should not be interpreted as evidence of no association or no potential benefit.

To rigorously evaluate whether these group differences represent statistically significant heterogeneity, formal interaction terms (e.g., smart BP monitor × gender, smart BP monitor × education) were introduced into the full multinomial logistic regression models. Formal interaction tests revealed weak evidence of interaction by gender (*p* = 0.097) and non-significant interactions for residence, education, and age (*p* > 0.400). Given the small sample sizes within specific strata (e.g., rural *n* = 302; adults aged 75 years or older *n* = 253) and the lack of strict statistical significance in the interaction terms, these variations should be interpreted cautiously as suggestive trends rather than definitive subgroup effects.

Given the exploratory nature of the subgroup analyses and the number of comparisons examined, some subgroup-specific differences may reflect chance variation; therefore, these findings should be interpreted as hypothesis-generating rather than confirmatory.

### 3.5. Robustness Checks and Sensitivity Analyses

To ensure the reliability of the longitudinal protective association of smart blood pressure monitors, a series of rigorous sensitivity analyses were conducted, addressing potential self-selection bias, baseline boundary effects, and confounding by unobserved digital literacy (Table 4).

To mitigate selection bias arising from observable differences between device users and non-users (e.g., higher income or urban residency predisposing individuals to technology adoption), we implemented Inverse Probability Weighting (IPW) using a propensity score approach (Model 2). After reweighting the pseudo-population to achieve covariate balance (see Table S4 for balance diagnostics), the estimated association between smart BP monitor use and transition into the Sensory Impaired class remained directionally consistent (RRR = 0.696). However, the association lost statistical significance at the 5% level (*p* = 0.162). After weighting, the confidence interval widened and the estimate was no longer statistically significant, suggesting that the core finding should be interpreted cautiously as a suggestive protective association rather than a definitive causal effect.

More specifically, the loss of statistical significance may reflect two complementary changes following weighting. First, the point estimate was attenuated toward the null relative to the corresponding unweighted specification in Model 1 (RRR from 0.626 to 0.696) after measured baseline differences between device users and non-users were balanced. Before weighting, appreciable imbalances were observed particularly in education, personal income, chronic disease burden, and internet use, whereas all post-weighting absolute standardized mean differences were below 0.10. This attenuation suggests that part of the stronger association in the unweighted model may have reflected measured differences in the baseline characteristics of users and non-users. A second consideration is that reweighting redistributed the contribution of individual observations and, together with robust sandwich variance estimation, may have modestly reduced statistical precision. The available weight diagnostics, including a 99th percentile of 5.21 for the unstabilized weights, did not indicate pronounced weight instability. Therefore, variance inflation alone is unlikely to fully explain the loss of statistical significance.

Second, to account for within-class variations in baseline disease severity, the continuous standardized Z-scores of all five intrinsic capacity domains were added to the base equation (Model 3). Even after adjustment for these continuous baseline functional measures, smart BP monitor use remained significantly associated with a lower relative risk of membership in the Sensory Impaired class (RRR = 0.581, 95% CI: 0.344–0.984, *p* = 0.043).

Finally, to rule out the possibility that the observed association was merely a proxy for general digital literacy, Model 4 restricted the analysis exclusively to the subpopulation of active internet users (N = 1273). Within this digitally literate cohort, the point estimate suggested a stronger protective association (RRR = 0.531), although the association was not statistically significant at the 5% level (*p* = 0.126), possibly reflecting, at least in part, the substantially reduced sample size. Collectively, these sensitivity analyses indicate that the direction of the association was generally consistent across alternative model specifications.

### 3.6. Mechanism Exploration: The Exploratory Pathway of Chronic Disease Stability

To explore the underlying pathways that may account for the protective association of smart BP monitor use, we employed an exploratory mediation framework to examine whether changes in chronic disease burden between 2020 and 2023 (ΔChronic Disease) mediated the association between baseline smart BP monitor use and the transition to impaired health states. Given the categorical nature of the outcome variable, we utilized the KHB (Karlson-Holm-Breen) method, which allows for the robust and unbiased decomposition of total effects into direct and indirect (mediated) effects within nonlinear models. However, this approach evaluates correlated longitudinal associations and does not establish strict sequential causality.

The KHB decomposition (Table 5) indicates a marginally significant total protective association of baseline smart BP monitor use against transitioning into the Sensory Impaired profile (Total effect = −0.510, *p* = 0.072). However, the indirect effect through ΔChronic Disease was not statistically significant (*p* = 0.256). This suggests that the protective trend of digital monitoring is not primarily mediated by a reduction in the aggregate count of chronic diagnoses over this three-year period. Instead, the benefits likely operate through direct pathways or other unmeasured daily management behaviors, such as improved medication adherence or timely symptomatic interventions that prevent functional exacerbation without necessarily altering the formal chronic disease count.

## 4. Discussion

### 4.1. Path Dependence of Intrinsic Capacity and the Vulnerability Trap

This study used a two-step latent profile transition framework to characterize longitudinal patterns of intrinsic capacity and to evaluate the association between smart health device use and follow-up IC profiles. A primary finding was the pronounced persistence of the Sensory Impaired profile: 62.79% of participants assigned to this profile at baseline were assigned to the corresponding profile again at follow-up. This robust persistence of sensory-locomotor impairment challenges the assumption that all early-stage functional decline is highly reversible, aligning with recent longitudinal evidence indicating that multidimensional organic impairments often co-occur and accumulate to form stable structural traps [15,16]. While existing literature frequently models IC or functional ability as a linear continuum [1,13,22], our profile-transition findings indicate that permanent organ-related impairments—particularly the concurrent decline of sensory and locomotor functions—create distinct vulnerability profiles with restricted recovery potential [23,24]. In contrast, the apparent reversibility of the Cognitively Impaired profile should be interpreted cautiously, as it may reflect survivor bias, regression to the mean, classification uncertainty, and practice effects rather than genuine clinical recovery. Furthermore, evidence suggests that the co-occurrence of dual sensory impairment and multimorbidity patterns substantially increases the odds of functional limitations [25,26]. The high persistence of the Sensory Impaired class in our cohort corroborates these findings, indicating that optimizing IC requires identifying these specific heterogeneous impairment patterns before they progress to permanent disability and further physiological decline [2,27].

### 4.2. Mechanism Deconstruction: The “Biological Buffer” of Digital Monitoring

Moving beyond descriptive trajectories, a central contribution of this study lies in identifying the targeted protective association of smart blood pressure (BP) monitor use. While the formal indirect effect through the stabilization of chronic disease burden was not statistically significant (*p* = 0.256), exploratory pathway analysis suggests a potential “biological buffer” mechanism. Rather than definitively altering the formal chronic disease count, these digital tools are hypothesized to facilitate anticipatory care by enabling precise, proactive interventions before cardiovascular events or systemic functional declines manifest [3,28]. IoT-based home monitoring and digital health interventions have been consistently associated with significant reductions in systolic and diastolic BP, alongside improved medication adherence among hypertensive populations [5,7,29]. If confirmed in future studies, smart BP monitoring may support disease-management processes that could potentially attenuate pathways linking cardiometabolic dysregulation to subsequent functional decline [20,30,31]. Additionally, the lack of significant longitudinal protection from other generic smart devices in our models corroborates the notion that the clinical utility of digital interventions for adults aged 75 years or older may be highly device-specific, relying heavily on active clinical management rather than passive data tracking [4,18,32].

### 4.3. Sociological Critique: Digital Divide vs. Compensatory Digital Dividend

From a sociological perspective, our subgroup analyses suggest possible sociodemographic variation that can be considered in relation to the competing digital-divide and compensatory-effect perspectives. A prominent concern in contemporary digital sociology is the Matthew effect, wherein digital economy developments and algorithms disproportionately benefit individuals with pre-existing high socioeconomic status (SES), thereby exacerbating health inequalities [8,10]. Conversely, our empirical results present suggestive trends aligning with the compensatory effect hypothesis. Although rigorous interaction tests yielded non-significant or marginally significant results (e.g., gender interaction *p* = 0.097), the associations between smart BP monitor use and a lower relative risk of sensory impairment appeared stronger among vulnerable groups, particularly women and older adults with primary education or below. If validated by future experimental research, these findings would suggest that accessible physiological monitoring might partially compensate for limited access to conventional healthcare resources [6,21]. This aligns with an emerging body of evidence demonstrating that digital infrastructure and targeted eHealth interventions may serve as potential compensatory tools under specific contexts, effectively reducing depressive symptoms and physical health disparities among lower-education cohorts [11,12,33].

However, this interpretation should be considered alongside an alternative explanation. The higher prevalence of electronic blood pressure monitor use among the Sensory Impaired group may also reflect confounding by indication. Older adults with greater disease burden, more frequent healthcare needs, or heightened health concerns may be more likely to adopt monitoring devices. Therefore, the observed higher adoption rate among vulnerable groups should not be interpreted solely as evidence of compensatory adoption, but may reflect a combination of increased health-management needs and potential benefits associated with targeted monitoring. Although we applied inverse probability weighting and adjusted for multiple baseline characteristics, residual confounding related to disease severity, healthcare-seeking behavior, family support, and prior health awareness cannot be completely excluded.

Beyond device availability itself, the realization of potential digital health benefits may also depend on users’ ability to effectively engage with digital resources. Literature suggests that critical eHealth literacy and social participation act as vital catalysts in this process, enabling lower-SES individuals to achieve health empowerment levels comparable to their advantaged counterparts [34,35,36]. The corresponding estimates among rural residents and adults aged 75 years or older were statistically non-significant and imprecise. Given the relatively small subgroup sizes and non-significant interaction tests, these findings cannot establish that the association is absent in these populations or identify the mechanisms underlying the observed numerical differences. Potential operational barriers and community-support needs should therefore be examined directly in adequately powered future studies [9,37].

Because the formal interaction tests were non-significant or only marginally significant, the present findings do not conclusively adjudicate between the Matthew-effect and compensatory-effect expectations. The subgroup patterns are more directionally compatible with the compensatory hypothesis, but they remain exploratory and may also reflect differential selection into device adoption.

### 4.4. Policy Implications and Limitations

These findings offer conceptual implications for healthy aging policies, though they must be strictly interpreted within the boundaries of our observational design and focus on a single physiological monitor within the CLASS cohort. Because the IPW adjustments attenuated the statistical significance of the protective trend, our conclusions are presented as associational hypotheses rather than definitive causal proofs. Therefore, rather than advocating for immediate large-scale policy mandates, our findings propose that the gradual integration of medical-grade, disease-specific digital interventions into community-based chronic care ecosystems warrants further quasi-experimental and randomized controlled testing [19,38]. To bridge the residual usage divide in rural and advanced-age populations, structural support systems—including age-friendly product redesigns and targeted digital literacy training—are essential to transform initial technological access into sustained health management habits [39,40,41].

Despite robust analytical strategies, several limitations warrant acknowledgment. First, relying on observational panel data precludes definitive causal confirmation. Although Inverse Probability Weighting (IPW) was employed to adjust for observed baseline differences (including income and internet usage), our estimates remain susceptible to unmeasured residual confounding. Crucial underlying factors—such as individual health literacy, broader health-seeking behaviors, structural access to conventional healthcare, unobserved family support dynamics, baseline hypertension severity, and medication adherence—could not be fully captured in the CLASS dataset. These unmeasured variables may simultaneously drive both the proactive adoption of smart devices and the maintenance of healthier capacity trajectories. Consequently, the observed relationships must be strictly interpreted as longitudinal protective associations rather than definitive causal effects.

Second, the measurement of intrinsic capacity partially depends on self-reported functions, which may introduce subjective bias. Future research should leverage objective, wearable continuous-sensing technologies and biomarkers [42,43] to further validate the precision and long-term equity of digital health management models.

Third, several methodological limitations of the profile-transition framework should be acknowledged. Our analytical framework employed a ‘classify-then-analyze’ approach relying on modal assignment (maximum posterior probabilities) for the 2020 and 2023 waves separately, rather than a jointly estimated full-information latent transition model. Longitudinal measurement invariance was neither formally tested nor imposed across waves; therefore, the observed cross-wave transition proportions may partly reflect changes in profile measurement or definition across waves, in addition to genuine changes in intrinsic capacity. Modal assignment also does not incorporate posterior classification uncertainty into the subsequent transition and regression analyses and may attenuate or otherwise bias the observed transition patterns and regression estimates. The direction and magnitude of this potential bias cannot be determined from the present analysis.

Fourth, construction of the final analytical cohort resulted in considerable attrition. The baseline comparison between included and non-retained baseline respondents indicated that the analytical cohort disproportionately represented urban and more highly educated older adults. Because the non-retained group included respondents who were lost to follow-up, died before follow-up, or had incomplete information on the study variables, the observed differences reflect the combined sample-selection process rather than complete-case exclusion alone. A dedicated multiple-imputation workflow integrated with wave-specific latent profile estimation and cross-wave profile alignment was not implemented. The complete-case component of the analysis was therefore conducted under a missing-at-random assumption, although this assumption cannot be empirically verified. Selection bias arising from attrition, mortality, and incomplete data cannot be excluded. The findings may therefore not generalize fully to rural residents, individuals with lower educational attainment, or other respondents not retained in the final analytical cohort.

Fifth, our interpretation of cognitive profile transitions is constrained by measurement variability and practice effects. The unusually high observed proportion of participants moving from the Cognitively Impaired profile at baseline to the Robust Capacity profile at follow-up may reflect practice effects, regression to the mean, survivor bias, and classification uncertainty rather than genuine clinical recovery. Future longitudinal studies should employ more strongly anchored cognitive measures, longitudinal item response theory (IRT) models, and objective cognitive assessments or biomarkers to better distinguish substantive functional change from measurement variability, classification uncertainty, and familiarity with repeated cognitive testing.

Sixth, our mechanism exploration regarding the “biological buffer” must be interpreted with distinct methodological caution. Valid longitudinal causal mediation requires strict sequential ignorability—meaning no unmeasured time-varying confounders influence both the mediator and the outcome—and clear temporal sequencing (e.g., a three-wave design). Because our data relies on a two-wave panel, there is an inherent temporal overlap: the proposed mediator (change in chronic disease burden from 2020–2023) and the outcome (2023 IC profile) are observed over the same window. Consequently, we cannot definitively rule out reverse causality (i.e., whether preserved capacity prevented new chronic diagnoses) or the influence of unmeasured time-varying health shocks. Furthermore, the KHB analysis did not yield a statistically significant indirect effect. Therefore, our KHB pathway analysis should be viewed strictly as an exploratory demonstration of correlated longitudinal associations rather than a sequenced causal mediation.

Finally, although the study and analytical plan were not preregistered, we have addressed concerns regarding researcher degrees of freedom by transparently reporting the full latent-class enumeration process, model-selection criteria, and statistical rationale for the final specification in Supplementary Table S3, thereby facilitating independent evaluation and replication.

## 5. Conclusions

In conclusion, this observational longitudinal study used latent profile transition analyses and propensity score weighting to examine the associations between smart physiological monitoring and intrinsic capacity (IC) trajectories. The Cognitively Impaired profile showed marked classification instability, which may reflect survivor bias, measurement variability, regression to the mean, classification uncertainty, and practice effects rather than true clinical recovery. However, targeted digital interventions—specifically smart BP monitor use—were associated with a lower relative risk of membership in the Sensory Impaired profile. The exploratory KHB analysis did not identify a statistically significant indirect effect through changes in chronic disease burden. Therefore, the pathway findings should not be interpreted as evidence of mediation.

Furthermore, our empirical evidence challenges the pervasive “digital divide” narrative and points toward a potential compensatory digital dividend, wherein highly vulnerable subpopulations—notably women and those with minimal educational attainment—may derive potential protective benefits. These findings suggest that future healthy aging strategies may benefit from considering disease-specific digital health interventions alongside broader digital inclusion initiatives. Further evidence from diverse populations and intervention studies is needed to determine their scalability and long-term effectiveness. Future research should leverage continuous-sensing wearables and objective biomarkers to further validate the precision and scalable equity of these digital health management models.

## Figures and Tables

**Table 1:** Baseline Sociodemographic and Health Characteristics of the Analytical Sample Stratified by Latent Intrinsic Capacity Profiles (Wave 2020, N = 2432).

Characteristics	Total Sample (N = 2432)	Class 1: Cognitively Impaired (*n* = 266, 10.9%)	Class 2: Sensory Impaired (*n* = 215, 8.8%)	Class 3: Robust Capacity (*n* = 1951, 80.2%)	*p*-Value
Intrinsic Capacity Indicators					
Cognition score (0–13)	11.33 (2.10)	7.11 (1.38)	10.08 (2.69)	12.04 (1.16)	<0.001
Psychological capacity (0–18)	11.73 (3.41)	10.89 (3.57)	10.29 (3.19)	11.89 (3.38)	<0.001
Sensory capacity (0–2)	1.92 (0.30)	2.00 (0.00)	0.93 (0.26)	2.00 (0.00)	<0.001
Locomotor function (2–16)	15.61 (1.34)	14.60 (2.78)	15.12 (2.03)	15.83 (0.74)	<0.001
Vitality (BMI)	23.49 (2.61)	23.85 (2.76)	24.35 (2.69)	23.33 (2.60)	<0.001
Sociodemographic Covariates					
Age (Years)	68.38 (4.70)	69.86 (5.21)	70.65 (5.63)	67.93 (4.40)	<0.001
Female (%)	50.58	53.38	54.88	49.77	0.230
Urban residents (%)	87.58	80.83	82.33	89.08	<0.001
Married and cohabiting (%)	83.51	74.81	81.86	84.88	<0.001
Educational attainment (%)					<0.001
-- Primary school and below	9.62	23.31	16.28	7.02	
-- Primary school	23.68	31.2	27.44	22.25	
-- Junior high school	42.39	33.08	34.88	44.49	
-- Senior high school and above	24.30	12.41	21.4	26.24	
Personal income (ln)	8.86 (1.06)	8.59 (1.25)	8.97 (1.19)	8.88 (1.01)	<0.001
Family and Health Covariates					
Household size	2.56 (1.19)	2.80 (1.30)	2.97 (1.33)	2.57 (1.22)	<0.001
Number of living children	1.78 (0.92)	1.91 (0.99)	1.77 (0.93)	1.76 (0.91)	0.045
Chronic conditions count	1.69 (1.34)	1.81 (1.43)	2.30 (1.79)	1.60 (1.25)	<0.001
Internet use habit (%)	52.34	25.19	39.07	57.51	<0.001
Social network activity (0–8)	3.21 (1.70)	3.02 (1.58)	3.18 (1.46)	3.25 (1.74)	0.082

Notes: Continuous variables are presented as mean (standard deviation); categorical variables are presented as percentages. Differences across the three latent classes were evaluated using analysis of variance (ANOVA) for continuous variables and Pearson’s chi-square tests for categorical variables. Intrinsic capacity (IC) indicators significantly differ across classes by definition of the Latent Profile Analysis (LPA) design. Baseline adoption rates for the eight specific smart health devices are detailed separately in Supplementary Table S5.

**Table 2:** Cross-Wave Transition Matrix of Assigned Intrinsic-Capacity Profiles, 2020–2023.

Baseline Latent Class (Wave 2020)	Assigned Profile in 2023, Row Percentage (%)			Total Baseline N
	Class 1: Cognitively Impaired	Class 2: Sensory Impaired	Class 3: Robust Capacity	
**Class 1: Cognitively Impaired**	10.53	2.26	87.22	266
**Class 2: Sensory Impaired**	6.51	62.79	30.7	215
**Class 3: Robust Capacity**	1.18	1.49	97.33	1951
**Total Sample Distribution (2023)**	2.67	6.99	90.34	2432

Notes: Values are row percentages based on modal profile assignments at the 2020 and 2023 waves. Each row shows the proportion of participants assigned to each follow-up profile among those assigned to a given baseline profile. These values represent observed transition proportions between assigned profiles rather than model-estimated latent transition probabilities.

**Table 3:** Multinomial Logistic Regression Predicting 2023 Latent Profile Membership (Reference Outcome = Class 3: Robust Capacity).

Predictors (Wave 2020)	Class 1: Cognitively Impaired		Class 2: Sensory Impaired	
	RRR (95% CI)	*p*-Value	RRR (95% CI)	*p*-Value
**Baseline Profile (Ref = Robust)**				
- Class 1: Cognitively Impaired	0.173 (0.089, 0.337)	<0.001	0.646 (0.269, 1.553)	0.329
- Class 2: Sensory Impaired	1.511 (0.683, 3.342)	0.308	96.397 (41.269, 225.169)	<0.001
**Smart Device Usage**				
- Electronic BP monitor	1.433 (0.775, 2.647)	0.251	0.557 (0.325, 0.955)	0.033
- Blood lipid tester	0.801 (0.339, 1.890)	0.612	1.640 (0.842, 3.191)	0.146
- Smart wristband/watch	1.242 (0.472, 3.267)	0.661	0.757 (0.383, 1.497)	0.424
- Smart health terminal	1.812 (0.653, 5.030)	0.254	1.240 (0.629, 2.443)	0.535
- Smart wheelchair	2.284 (0.829, 6.293)	0.110	1.251 (0.456, 3.437)	0.664
- Infrared monitor/camera	0.564 (0.083, 3.861)	0.560	1.241 (0.353, 4.367)	0.736
- Smart sleep monitor	1.108 (0.254, 4.830)	0.892	0.488 (0.154, 1.549)	0.223
- Audiobooks/Smart audio	0.835 (0.189, 3.691)	0.812	1.812 (0.861, 3.813)	0.117
**Covariates**				
- Age (Years)	1.168 (1.104, 1.235)	<0.001	1.074 (1.013, 1.139)	0.017
- Male (Ref = Female)	0.723 (0.410, 1.274)	0.262	0.495 (0.310, 0.790)	0.003
- Urban resident	0.218 (0.093, 0.511)	<0.001	0.735 (0.353, 1.531)	0.411
- Married and cohabiting	0.666 (0.360, 1.232)	0.196	0.808 (0.439, 1.486)	0.493
- Educational attainment	1.011 (0.725, 1.409)	0.949	1.017 (0.779, 1.327)	0.903
- Personal income (ln)	1.496 (1.077, 2.079)	0.016	0.831 (0.616, 1.122)	0.227
- Household size	0.979 (0.756, 1.268)	0.874	1.028 (0.831, 1.272)	0.797
- Living children count	0.981 (0.735, 1.309)	0.894	0.888 (0.651, 1.212)	0.453
- Chronic conditions count	1.077 (0.878, 1.320)	0.478	1.108 (0.964, 1.274)	0.150
- Internet use habit	0.626 (0.271, 1.444)	0.272	1.412 (0.815, 2.446)	0.219
- Social network score	0.759 (0.602, 0.957)	0.020	0.988 (0.862, 1.133)	0.865

Notes: N = 2432. Pseudo R^2^ = 0.434, Wald χ^2^(42) = 601.58, *p* < 0.001. RRR = Relative Risk Ratios; CI = Confidence Interval. The dependent variable is the latent profile membership in Wave 2023. Class 3: Robust Capacity serves as the base outcome. RRR values below 1.0 indicate a protective effect (reduced risk of transitioning into the impaired class), whereas values above 1.0 denote an increased risk factor. The exceptionally high RRR (96.397) and wide confidence interval for baseline Class 2 predicting 2023 Class 2 indicate quasi-complete separation and should be interpreted cautiously. A supplementary Firth penalized binary logistic regression for the contrast between the Sensory Impaired and Robust Capacity profiles yielded a directionally consistent but statistically non-significant estimate for smart BP monitor use (OR = 0.605, 95% CI: 0.359–1.020, *p* = 0.059). Therefore, uncertainty related to sparse data and separation cannot be completely excluded.

**Table 4:** Relative Risk Ratios (RRR) for the Association between Smart BP Monitor Use and Transition into the Sensory Impaired Class.

Models	Estimation Strategy	RRR (95% CI)	*p*-Value
**Model 1**	Baseline Multinomial Logit (Reduced Covariates)	0.626 (0.378, 1.036)	0.069
**Model 2**	Inverse Probability Weighting (IPW)	0.696 (0.419, 1.156)	0.162
**Model 3**	Controlling for Continuous Baseline Z-scores	0.581 (0.344, 0.984)	0.043
**Model 4**	Restricted Subsample (Active Internet Users Only)	0.531 (0.236, 1.196)	0.126

Notes: N = 2432 for Models 1–3; N = 1273 for Model 4. The dependent variable is the latent profile membership in Wave 2023, with Class 3: Robust Capacity serving as the reference outcome. Values reported are the Relative Risk Ratios (RRR) specific to predicting the transition into Class 2: Sensory Impaired. RRR values below 1.0 indicate a lower relative risk of membership in the Sensory Impaired profile relative to the Robust Capacity profile. Model 1 explicitly includes the full set of baseline covariates: age, gender, urban residency, marital status, education level, log-income, household size, living children count, chronic conditions count, internet usage, and social network score. Models 2–4 also adjust for these same covariates unless dynamically modified by the respective estimation strategy.

**Table 5:** KHB Pathway Analysis: Decomposing the Association between Smart BP Monitor Use and Transition to the Sensory Impaired Class.

Effect Component	Coefficient (Log-Odds)	Robust Std. Err.	z-Value	*p*-Value	95% CI
Total Effect (Reduced)	−0.510	0.284	−1.80	0.072	(−1.067, 0.047)
Direct Effect (Full)	−0.491	0.285	−1.72	0.085	(−1.050, 0.068)
Indirect Effect (Diff)	−0.019	0.017	−1.14	0.256	(−0.052, 0.014)

Notes: Estimates were derived using the KHB (Karlson-Holm-Breen) logit method to correctly decompose effects for a categorical outcome. The outcome presented is the transition to Class 2 (Sensory Impaired) relative to Class 3 (Robust). The coefficients are reported as Log-Odds, where negative values indicate a protective association (reduced risk). The formal indirect effect reflects the pathway through the stabilization of chronic disease burden (ΔChronic Disease). The models controlled for baseline latent class, age, gender, urban residency, marital status, education, log-income, baseline chronic count, internet usage, and social network score. Robust SE denotes the robust standard error used to calculate the corresponding z-statistic, *p*-value, and 95% confidence interval.

## Data Availability

The data analyzed in this study are publicly accessible upon formal application. The data were obtained from the China Longitudinal Aging Social Survey (CLASS), administered by the National Survey Research Center at Renmin University of China. Researchers may apply for access to the dataset through the official CLASS website: http://class.ruc.edu.cn/. Access is subject to formal application and completion of the required data use agreement.

## Supplementary Materials

The following supporting information can be downloaded at https://www.mdpi.com/article/10.3390/healthcare14152254/s1: Table S1: Detailed Cross-Wave Sample Selection and Symmetric Attrition Matrix (CLASS 2020–2023); Table S2: Detailed Variable Definitions and Operationalization; Table S3. Fit Indices for Latent Profile Analysis Models Exploring Intrinsic Capacity (N = 2432); Table S4. Covariate Balance Diagnostics Before and After Inverse Probability Weighting (IPW); Table S5. Baseline Adoption Rates of Eight Smart Health Devices Stratified by Latent Intrinsic Capacity Profiles (Wave 2020, N = 2432); Table S6. Baseline Characteristics of Respondents Included in the Final Analytical Cohort versus Those Excluded Due to Complete-Case Attrition.
