# Supplementary material for: Digital Divide or Compensatory Dividend? The Longitudinal Impact of Smart Health Monitoring on Intrinsic Capacity Trajectories
Source: Healthcare (Basel). 2026 Jul 23;14(15):2254. doi: 10.3390/healthcare14152254 (PMC13465248; doi:10.3390/healthcare14152254)
Supplement: Supplementary file 1 [file healthcare-14-02254-s001.zip › healthcare-4377932-supplementary.pdf]

Table S1. Detailed Cross-Wave Sample Selection and Symmetric Attrition Matrix (CLASS 2020 – 2023).

Selection Stage and Exclusion Criteria	2020 Wave Rows (N)	2023 Wave Rows (N)	Total Pooled Rows (N)	Matched Individuals (n)
Initial Pool of CLASS Respondents	11,395	11,670	23,065	—
Stage 1: Longitudinal Core Alignment				
- Less: Lost to follow-up or deceased by 2023	-4,369	—	-4,369	—
- Less: Newly recruited rotation sample in 2023	—	-4,644	-4,644	—
Base Longitudinal Tracking Cohort	7,026	7,026	14,052	7,026
Stage 2: Symmetric Casewise Attrition Filtering				
- Less: Symmetrical removal of incomplete cases (a)	-4,594	-4,594	-9,188	-4,594
**Final Analytical Sample (Strongly Balanced Panel)**	**2,432**	**2,432**	**4,864**	**2,432**

Notes: Data are compiled from the 2020 and 2023 longitudinal waves of the China Longitudinal Aging Social Survey (CLASS). (a) Symmetric Casewise Attrition Filtering: To satisfy LTA requirements for paired transition probabilities (Pattern 11), a strict person-level listwise deletion was enforced. If a respondent incurred an item non-response or an invalid measurement in either 2020 or 2023, the individual's records across both waves were simultaneously pruned to maintain panel symmetry. For transparency, the raw item-level missingness flags detected within the 14,052 baseline tracking rows prior to the symmetric cross-wave deletion included: Missing values in Intrinsic Capacity (IC) dimensions (93 rows); Item non-response in primary baseline covariates, predominantly personal income (4,700 rows); Missing values in supplementary social and family metrics, including children alive and social network scores (358 rows). These items are non-mutually exclusive as an individual may exhibit missingness across multiple variables simultaneously.

Table S2. Detailed Variable Definitions and Operationalization.

Category	Variable Name	Operationalization / Measurement
**Dependent Var**	Latent Class (2023)	Derived via LPA based on standardized Z-scores of 5 IC domains (Cognition, Psychological, Sensory, Locomotor, Vitality).
**Independent Var**	Smart BP Monitor	Items: Baseline self-reported ownership and use of an electronic blood pressure monitor in the 2020 CLASS wave.<br>Scoring: Responses were coded as 1 = yes and 0 = no.<br>Interpretation: This binary variable was the focal exposure in the IPW, subgroup, robustness, and pathway analyses.
**Intrinsic Capacity**	Cognition	Items: Cognitive performance was assessed using temporal-orientation questions (e.g., current year, month, date, day of the week, and season), mathematical calculation tasks, and a delayed word-recall test from the CLASS questionnaire.<br>Scoring: One point was assigned for each correct response, and the item scores were summed.<br>Interpretation: The total score ranged from 0 to 13, with higher scores indicating better cognitive function.
	Psychological	tems: 9 items from the CLASS psychological module (adapted from the CES-D scale) assessing recent depressive, anxious, and positive psychological functioning.<br>Scoring: Negatively worded items were reverse-coded, and all items were summed.<br>Interpretation: The total score ranged from 0 to 18, with higher scores indicating better psychological capacity.
	Sensory	Items: Two self-reported items assessing visual and hearing function.<br>Scoring: Each preserved sensory function was coded as 1 and impairment as 0. Interpretation: Scores ranged from 0 to 2, with higher scores indicating better sensory capacity.
	Locomotor	Items: 8 Activities of Daily Living (ADL) and Instrumental Activities of Daily Living (IADL) items from the CLASS mobility-function module (e.g., dressing, bathing, walking, and doing household chores).<br>Scoring: Item responses were coded in the same positive direction and summed. Interpretation: Scores ranged from 2 to 16, with higher scores indicating better locomotor function.

	Vitality	Items: Self-reported body weight and height.<br>Scoring: BMI was calculated as weight in kilograms divided by height in meters squared. Interpretation: BMI was used as a survey-based proxy for the vitality domain and standardized before latent-profile analysis.
**Socio-demographics**	Age	Continuous variable (Years).
	Gender	Categorical (1 = Male, 0 = Female).
	Residence	Categorical (1 = Urban, 0 = Rural).
	Education	Categorical (1 = Primary & below; 2 = Primary; 3 = Junior high; 4 = Senior high & above).
	Income	Natural log-transformed annual personal income (ln).
**Health & Lifestyle**	Chronic Conditions	Count of self-reported doctor-diagnosed chronic illnesses (e.g., hypertension, diabetes, heart disease).
	Internet Usage	Binary (1 = Usage habit, 0 = None).
	Social Network	Sum score of frequency of interaction with family, friends, and community (Range: 0–8).

Table S3. Fit Indices for Latent Profile Analysis Models Exploring Intrinsic Capacity (N = 2,432).

Number of Classes	Log-Likelihood	Free Parameters	AIC	BIC	Entropy	Min Class Size (%)
2-Class Model	-16556.76	16	33145.51	33238.25	0.925	18.30
3-Class Model	-14153.19	19	28344.38	28454.51	0.941	8.84
4-Class Model	-13484.55	25	27019.11	27164.02	0.750	1.81

Notes: AIC = Akaike Information Criterion; BIC = Bayesian Information Criterion. The 5-class model failed to converge, indicating severe model over-extraction. Although information criteria slightly favored the 4-class model, its classification quality deteriorated substantially (Entropy dropped to 0.750), and it produced a fragmented latent class comprising only 1.81% of the sample, violating minimum class size viability. Therefore, the 3-class model was selected as the optimal taxonomy, balancing empirical fit, high classification quality (Entropy = 0.941), and theoretical interpretability. The Lo–Mendell–Rubin adjusted likelihood-ratio test and the bootstrapped likelihood-ratio test were not available under the Stata/SE 17.0 gsem estimation workflow and were therefore not used as class-enumeration criteria. Model selection was based on the combined consideration of AIC, BIC, entropy, minimum class prevalence, convergence stability, and substantive interpretability.

Table S4. Covariate Balance Diagnostics Before and After Inverse Probability Weighting (IPW)

Covariates (Baseline 2020)	Raw Standardized Difference (SMD)	Weighted Standardized Difference (SMD)	Variance Ratio (Raw)	Variance Ratio (Weighted)
Age	-0.0829	-0.0007	0.942	0.947
Male	-0.0874	0.0067	0.998	1.000
Urban resident	0.1027	-0.0061	0.791	1.012
Married	0.1119	-0.0050	0.817	1.009
Education Level	0.2251	-0.0097	0.932	1.042
Personal Income (ln)	0.3598	0.0022	0.930	1.078
Chronic Conditions Count	0.3918	0.0288	1.449	1.051
Internet Usage	0.3554	-0.0014	0.976	1.000

Notes: IPW utilizing Average Treatment Effect (ATE) unstabilized weights. A Standardized Mean Difference (SMD) close to 0 (typically < 0.10) and a variance ratio close to 1.0 indicate optimal balance between the treated (smart BP monitor users) and control (non-users) groups. Following weighting, all covariates demonstrated absolute SMDs well below the 0.10 threshold, confirming a highly balanced pseudo-population.

Table S5. Baseline Use Rates of Eight Smart Health Devices Stratified by Latent Intrinsic Capacity Profiles (Wave 2020, N = 2,432)

Characteristics	Total Sample (N = 2,432)	Class 1: Cognitively Impaired (n = 266, 10.9%)	Class 2: Sensory Impaired (n = 215, 8.8%)	Class 3: Robust Capacity (n = 1,951, 80.2%)	p-value
Electronic blood pressure monitor	53.47	54.89	72.56	52.02	< 0.001
Blood lipid tester	17.56	11.28	18.14	16.56	< 0.001
Smart wristband/watch	16.76	7.14	22.79	17.22	< 0.001
Smart health terminal	10.49	5.26	10.7	11.58	< 0.001
Smart wheelchair	3.17	5.64	6.05	2.67	< 0.001
Infrared monitor/camera	3.76	2.26	4.19	2.51	< 0.001
Smart sleep monitor	4.38	1.5	5.58	3.02	< 0.001
Audiobooks/Smart audio	5.69	2.26	12.09	5.54	< 0.001

Notes: Values represent the percentage of older adults reporting the use of each respective smart health device at baseline. Differences across the three latent profiles were evaluated using Pearson's chi-square tests. Significant p-values (< 0.05) indicate unequal distribution of device use across the health states.

Table S6. Baseline Characteristics of Respondents Included in the Final Analytical Cohort versus Those Excluded Due to Complete-Case Attrition.

Characteristics	Included Respondents (n = 2,432)	Excluded Respondents (n = 8,963)	p-value
**Socio-demographic Covariates**			
Age (Years), mean (SD)	68.38 (4.70)	72.46 (6.77)	< 0.001
Female (%)	50.58	49.29	0.261
Rural resident (%)	12.42	53.81	< 0.001
Married and cohabiting (%)	83.51	73.16	< 0.001
**Educational attainment (%)**			< 0.001
— Primary school and below	9.62	32.54	
— Primary school	23.68	40.33	
— Junior high school	42.39	19.95	
— Senior high school and above	24.30	7.17	
Personal income (ln), mean (SD)	8.86 (1.06)	8.87 (1.07)	0.822

**Family and Health Covariates**			
Chronic conditions count, mean (SD)	1.69 (1.34)	1.61 (1.39)	0.012
Internet use habit (%)	52.34	20.57	< 0.001
Social network activity, mean (SD)	3.21 (1.70)	3.24 (1.83)	0.577
Intrinsic Capacity Indicator			
Baseline Cognition score, mean (SD)	11.33 (2.10)	10.44 (2.94)	< 0.001

Notes: Continuous variables are presented as mean (standard deviation); categorical variables are presented as percentages. P-values were derived from independent sample t-tests for continuous variables and Pearson's chi-square tests for categorical variables. The "Included Respondents" (n = 2,432) represent the strongly balanced panel cohort utilized in the primary LTA and regression models, characterized by complete data across the 2020 and 2023 tracking waves. The "Excluded Respondents" (n = 8,963) consist of individuals removed due to loss to follow-up or item non-response across either wave. While complete-case attrition disproportionately affected rural and lower-education respondents, no significant differences were observed regarding gender, personal income, or social network activity.
